# Physicochemical Principles Governing the Intrinsically Disordered Salivary Peptide Histatin 5: Ensemble Structure, Electrostatics, and Environmental Responsiveness

**DOI:** 10.1021/acs.jpcb.6c00591

**Published:** 2026-03-30

**Authors:** Oskar Svensson, Samuel Lenton, Marie Skepö

**Affiliations:** † Division of Computational Chemistry, Department of Chemistry, Science for Life Laboratory, Lund University, P.O. Box 124, SE-221 00 Lund, Sweden; ‡ NanoLund, Lund University, Box 118, SE-221 00 Lund, Sweden; § Department of Pharmacy, Faculty of Health and Medical Sciences, University of Copenhagen, 2100 Copenhagen, Denmark

## Abstract

Histatin 5 (Hst5) is a histidine-rich intrinsically disordered protein (IDP) whose biological function arises from a highly heterogeneous conformational ensemble and environmental sensitivity. Unlike structured antimicrobial peptides that rely on persistent secondary motifs, Hst5 remains disordered across a wide range of conditions, enabling continuous adaptation to changes in pH, ionic strength, metal-ion concentration, macromolecular crowding, and interfaces such as membranes and mineral surfaces. Recent advances in small-angle X-ray scattering (SAXS), neutron-based methods, and surface-sensitive techniques, combined with computer simulations, have allowed quantitative characterization of Hst5′s ensemble structure, thermodynamics, and interactions. This work synthesizes current understanding of how intrinsic disorder, charge regulation, histidine chemistry, and multiscale interactions govern Hst5 behavior. Beyond its biological relevance, Hst5 has emerged as a benchmark system for elucidating general physicochemical principles of IDPs and for testing integrative and machine-learning approaches that map sequence features to ensemble architecture.

## Graphical abstract

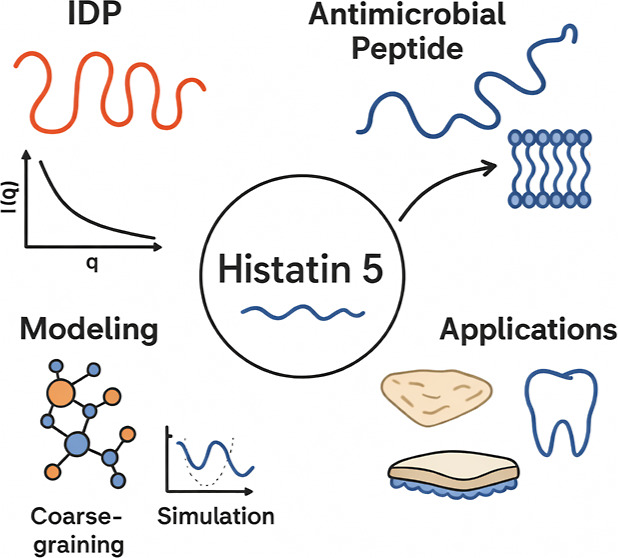

## Introduction

Intrinsically disordered proteins and peptides (IDPs) are central to many biological processes, functioning through dynamic, heterogeneous conformational ensembles rather than well-defined folded structures.^1–3^ Over the past two decades, advances in scattering techniques, spectroscopy, and molecular simulations have transformed our understanding of these systems, highlighting the critical roles of electrostatics, temperature, solvent-mediated interactions, and charge regulation in shaping ensemble behavior.^4–9^

Much of our work has focused on the salivary IDP Histatin 5 (Hst5) as a model system to elucidate general physicochemical principles of disordered peptides. In vivo Hst5 functions as an antifungal by contributing to innate defense against Candida in the oral cavity.^10^ Many antimicrobial peptides adopt defined secondary structures upon membrane binding, yet Hst5 retains its disordered conformation even at interfaces, making it an ideal probe for understanding ensemble thermodynamics, solvent-mediated interactions, and the influence of local backbone preferences on global dimensions.^11^

The responsiveness of Hst5 to pH, ionic composition, metal ions, macromolecular crowding, and surfaces illustrates the versatility conferred by intrinsic disorder.

In this feature article, we aim to provide a comprehensive synthesis of the physicochemical principles governing the IDP Hst5. By integrating experimental measurements, including small-angle X-ray scattering (SAXS), neutron-based methods, and surface-sensitive techniques, with atomistic, coarse-grained, and ensemble-based simulations, we highlight how sequence composition, electrostatics, histidine-mediated charge regulation, and local backbone preferences collectively shape Hst5′s heterogeneous conformational ensemble.

We also examine how environmental factors, including pH, ionic strength, multivalent ions, macromolecular crowding, and interfaces, modulate its structure and function. Beyond Hst5′s biological relevance, this article illustrates how IDPs can serve as benchmark systems for testing theoretical models, multiscale simulations, and machine-learning approaches that connect sequence features to ensemble behavior, providing generalizable insights into the interplay between disorder, electrostatics, and functional adaptability in peptides. Figure 1 illustrates the diverse conformational ensemble of Hst5, showing how its structures vary with radius of gyration (*R_g_* ) and how an increasing *R_g_* corresponds to more extended particle shapes, as evidenced by the scattering profiles, Kratky plots, and pair distance distributions. The experimental and computational techniques that have been used to characterize Hst5 are given in Table 1.

**Figure 1:**
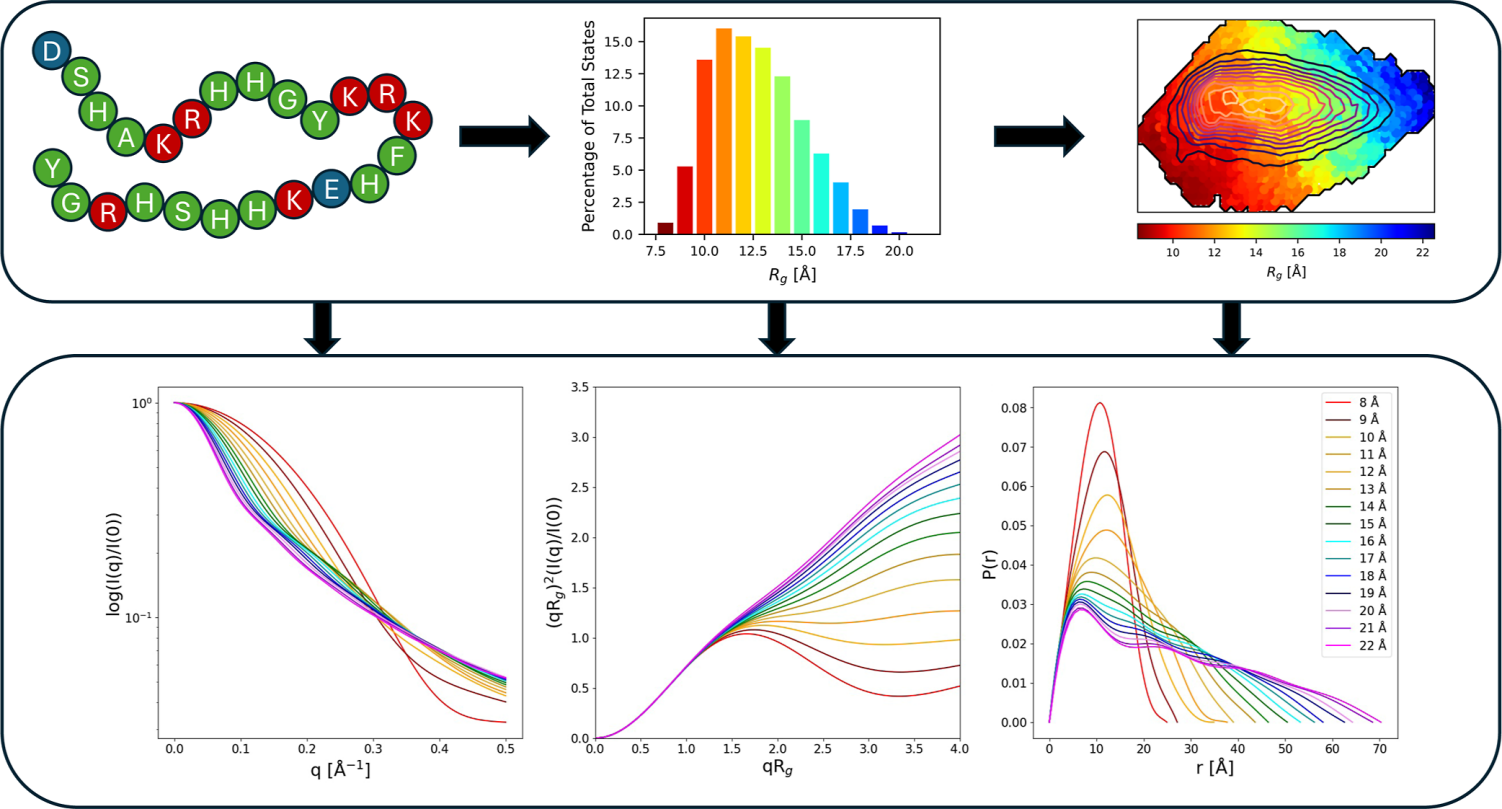
Histatin 5 (Hst5) provides a good system for modeling intrinsically disordered proteins; its diverse conformational ensemble is shown herein. Arising from a combination of intramolecular and intermolecular interactions, Hst5's varying states may be classified using the radius of gyration (*R_g_*), although this is merely a simplification of its true conformational landscape, which is further detailed using decomposed, simulated, small-angle X-ray scattering spectra (bottom left). Each spectrum describes the scattering intensity (I­(q)) as a function of the scattering angle (q), which has been determined from simulated structures that populate specific Note, especially, the Kratky plots (bottom middle), which show how the average particle shape becomes more extended with higher *R_g_* .Adapted with permission from ref 13 . Copyright 2024 American Chemical Society under license CC-BY 4.0.

**Table 1:** Experimental and Computational Techniques Used to Characterize Histatin 5

technique/method	observable/measured property	key insights for Hst5	representative references
Small-Angle X-ray Scattering (SAXS)	Radius of gyration (*R*_g_), pair-distance distribution P(r), ensemble dimensions	Hst5 remains disordered in solution, expanded ensemble, comparison across Histatin family	^12–16^
Dynamic Light Scattering (DLS)	Hydrodynamic radius, diffusion	Ensemble size, crowding effects	^17–19^
Neutron Scattering (QENS, NR)	Translational diffusion, membrane interaction, hydration	Diffusion slowdown under crowding, adsorption to membranes, orientation of ensembles	^19,20^
Nuclear Magnetic Resonance (NMR)	Chemical shifts, J-couplings, relaxation rates	Local backbone dynamics, PPII (polyproline II) propensity, residue-specific flexibility	^21,22^
Molecular Dynamics (MD) Simulations (all-atom)	Conformational ensemble, PPII content, *R*_g_, secondary structure propensities	Effect of force fields, water models, local stiffness, ensemble heterogeneity	^13,22–25^
Coarse-Grained (CG) Simulations/Monte Carlo	Ensemble dimensions, adsorption, oligomerization, crowding	Long-time scale behavior, surface interactions, high-concentration effects	^14,15,26–31^
Integrative/Ensemble Modeling (MD + SAXS/NMR)	Experimentally restrained ensembles	Ensemble refinement, validation against scattering data, sequence-ensemble mapping	^12,18,22,32–35^
Surface-Sensitive lab-based Techniques (QCM-D, Ellipsometry, Langmuir Monolayers)	Adsorption kinetics, layer thickness, surface coverage	pH- and ion-dependent adsorption, interaction with membranes and mineral surfaces	^9,20,36–38^
Metal Ion Binding/Spectroscopy	Zn^2+^/Cu^2+^ binding modes, stoichiometry, reversible oligomerization	Dynamical oligomerization, modulation of ensemble properties, charge regulation	^21,39–42^
Thermodynamic and Charge Regulation Analysis	Net charge vs pH, protonation states, ensemble energy	pH-responsive charge regulation, electrostatic ensemble tuning	^4,5,33,43^

## Conformational Ensembles

### Ensemble Properties

Hst5 populates a broad and highly heterogeneous conformational ensemble, characteristic of an expanded, self-avoiding polyelectrolyte chain. SAXS, dynamic light scattering (DLS), and molecular simulations consistently show that Hst5 lacks persistent secondary or tertiary structure, with R_g_ exceeding those expected for neutral random coils of comparable length, as is showcased in Figure 1.^17,28^

Comparative studies across the histatin family reveal that Hst5 is more extended and conformationally diverse than Histatin 1 (Hst1) or Histatin 3 (Hst3), a behavior attributable to sequence-specific charge patterning and the strategic placement of histidine residues.^12^ These observations align with previously determined *R*_g_ trends seen in IDPs, where the net charge per residue and long-range electrostatic repulsion dominate ensemble dimensions. Hst5 exhibits effective Flory exponents exceeding those of neutrally charged IDPs, positioning it near the upper bound of expanded disordered behavior.^27^

Computational studies further highlight the sensitivity of the ensemble to force-field selection and solvent representation. IDP-specific force fields and dispersion-optimized water models, such as TIP4P-D, reproduce ensemble dimensions, local stiffness, and polyproline II (PPII) propensity more accurately than conventional models.^22–24^ Macromolecular crowding has a modest influence on overall expansion, consistent with SAXS and neutron scattering measurements, indicating that Hst5 retains its extended character even under biologically relevant crowded conditions.^15,19^

These results show how sequence composition, electrostatic patterning, and solvent interactions define Hst5′s ensemble and its functional interactions with solid and membrane interfaces.

### Local Structure and Polyproline II Propensity

Although globally disordered, Hst5 exhibits pronounced local structure in the form of PPII helices. These PPII states are stabilized by favorable peptide–water interactions and impose local stiffness, effectively increasing the chain’s persistence length and modulating flexibility.^33^ Accurate simulation of these populations requires careful treatment of protein–water dispersion interactions, highlighting the need for TIP4P-D water models and IDP-tuned force fields to capture realistic local conformational preferences.^22,23^

Comparative analyses of flexible peptides and full-length IDPs reveal that capturing PPII content is essential for realistic modeling of the conformational ensemble.^22^ This emphasizes the delicate balance between local backbone order and global chain disorder in Hst5 and other highly charged IDPs; as is further contextualized in Figure 2 for Hst5 Local structural motifs, such as PPII helices, couple to long-range electrostatic interactions and sequence-specific charge patterning, collectively influencing overall ensemble expansion, flexibility, and responsiveness to environmental changes.

**Figure 2:**
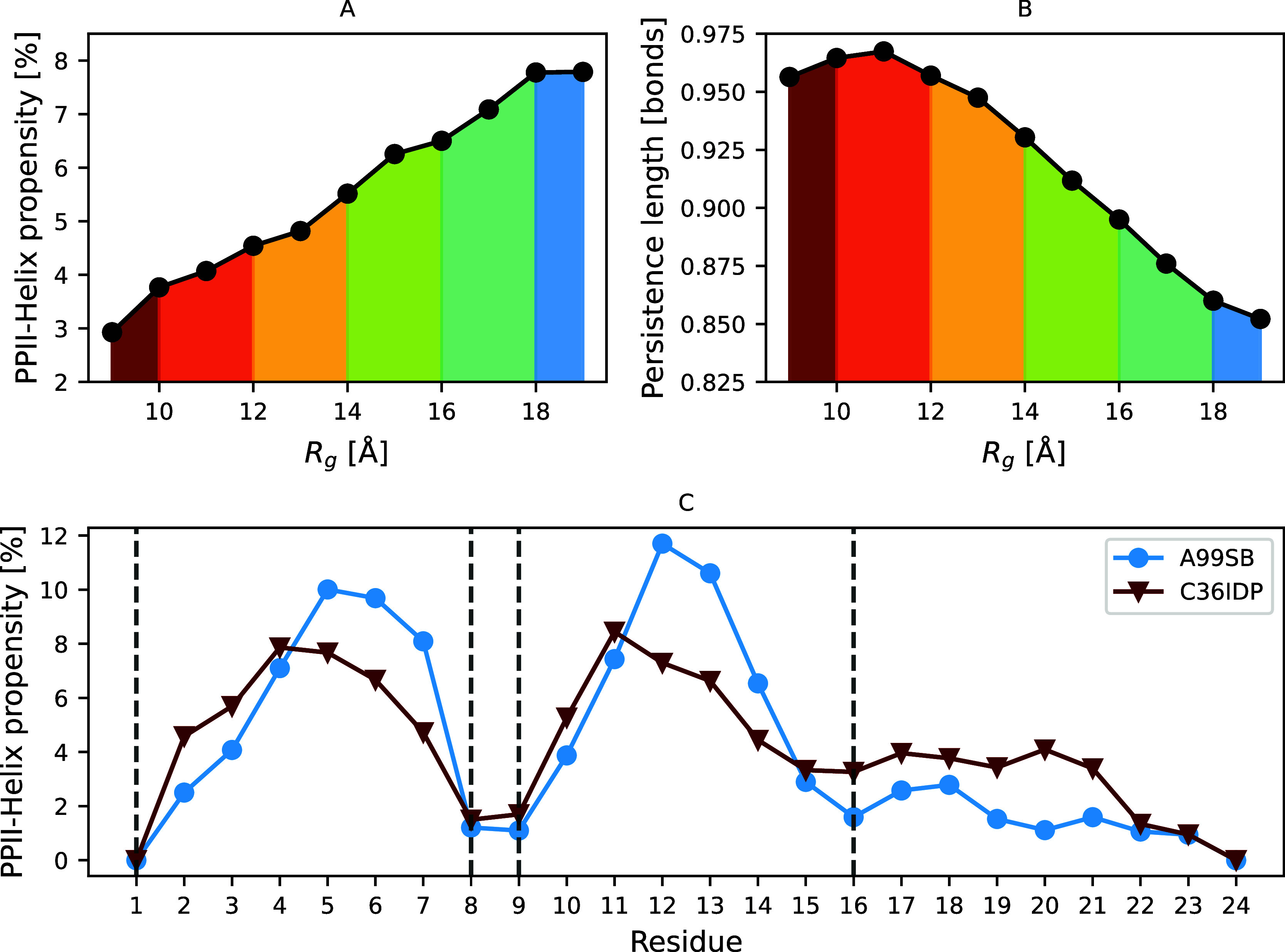
Global expansion and local backbone structure of Histatin 5 (Hst5) conformational ensemble. Hst5's expandend radius of gyration (*R_g_*) is directly connected to polyproline II (PPII) helix occurrence (A). Variation of the persistence length, as a function of *R_g_,* further indicates increases in chain stiffness (B). PPII helices are transient and local elements which can be readily modeled, altough, the choice of force field and water model is paramount (C). Adapted with permission from ref 22 . Copyright 2021 American Chemical Society under license CC-BY 4.0.

## Environmental Modulation and Charge Regulation

Hst5 exhibits environmental responsiveness, primarily governed by histidine protonation equilibria. Because the p*K*_a_ values of histidine residues lie close to physiological pH, modest changes in pH induce substantial variations in Hst5 net-charge and consequently intrachain electrostatic repulsion between charged amino acids ­(Figure 4A). These protonation-dependent charge fluctuations broaden the conformational ensemble, enhancing interactions with anionic partners and modulating the free energies of adsorption and translocation without the need for defined structural transitions.^4,9,37,38^ Charge regulation theory provides a quantitative framework for understanding this behavior, highlighting how dynamic adjustments in residue protonation contribute directly to ensemble energetics and binding equilibria.^4^ Furthermore, the adaptability of Hst5 in response to changing environmental conditions may enhance its anticandidal activityin the oral cavity.^39^ Temperature-dependent simulations further reveal polymer-like behavior: ensemble shifts occur gradually rather than through cooperative transitions, consistent with experimental observations of IDP flexibility and responsiveness.^33^

**Figure 3:**
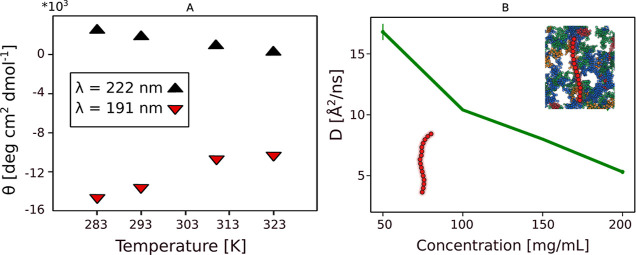
Effect of temperature and crowding on Histatin 5 (Hst5) structure and dynamics. (A) CD data showing the change of ellipticity at the indicated wavelengths and temperatures, indicating the destabilization of PPII structure with increasing temperature. (B) QENS data showing the decrease in the diffusion coefficient of Hst5 with increasing crowding. Panel A is reprinted with permission from ref 33. Copyright 2019 American Chemical Society under Standard ACS AuthorChoice/Editors’ Choice Usage Agreement, and panel B is reprinted with permission from ref 19. Copyright 2022 American Chemical Society license under CC-BY 4.0.

**Figure 4:**
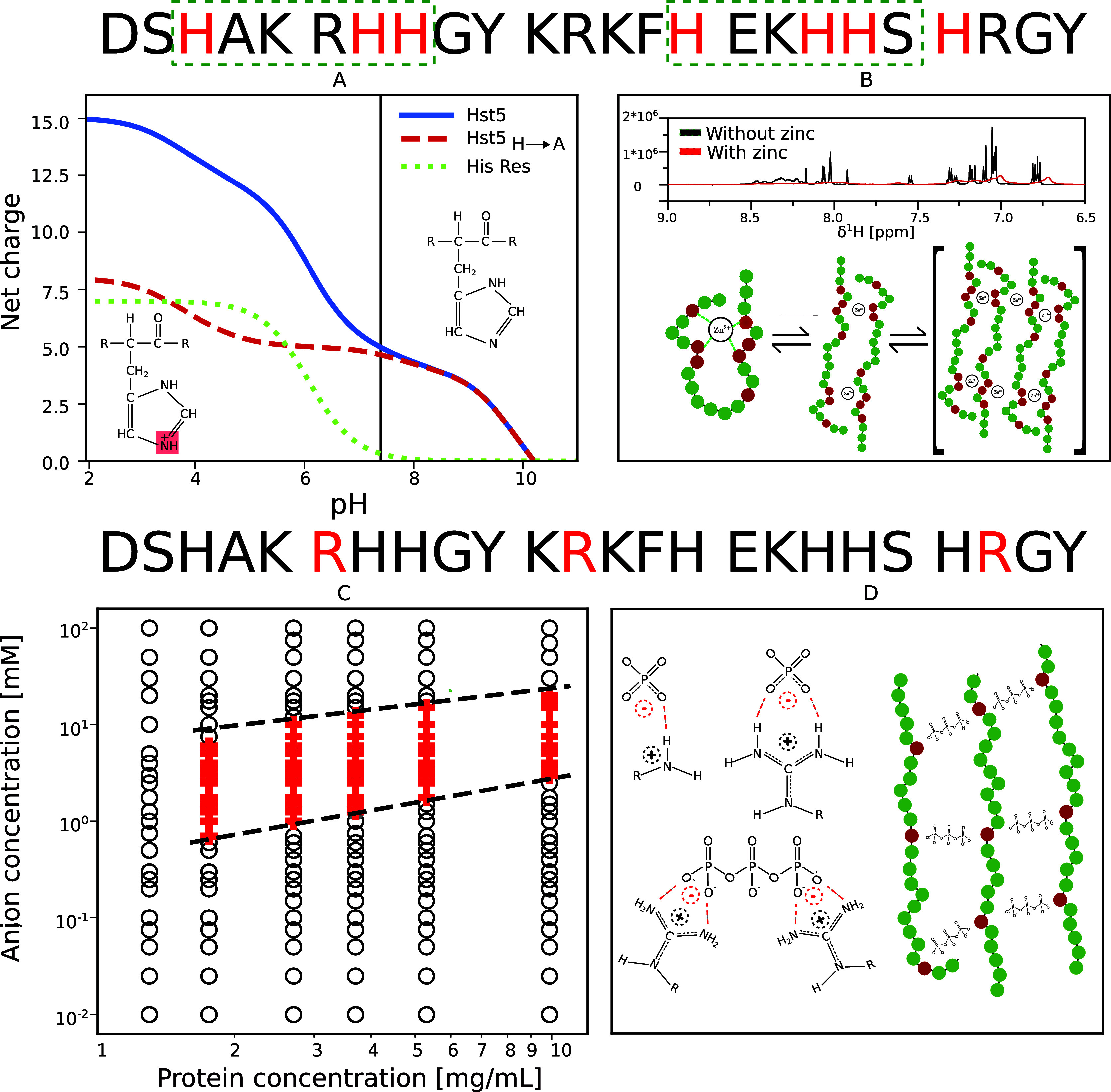
Charge regulation and multivalent interactions govern the solution properties of Histatin 5 (Hst5) (A) pH-dependent net charge of Hst5 compared with a histidine-to-alanine variant, highlighting histidine contributions. (B) Zinc-induced dynamic oligomerization observed by NMR and a schematic depiction of highly dynamic oligomers. (C) Phase diagram of Hst5 in the presence of tripolyphosphate. (D) Conceptual illustration of polyphosphate-driven reentrant condensation. Panel B is reproduced with permission from ref 21. Available under a CC-BY 4.0 license. Copyright 2019 Cragnell et al. Panel C reproduced with permission from ref 43. Copyright 2021 American Chemical Society.

Macromolecular crowding introduces additional modulation. Translational diffusion is slowed with increasing crowding conditions, see Figure 3. Note that the measured diffusion coefficients reflect the local environment experienced by the peptide under crowded conditions rather than solely bulk viscosity effects. SAXS-derived *R*_g_ values indicate mild ensemble compaction; yet the overall heterogeneity and global expansion of Hst5 remain largely preserved.^17,19^ These findings emphasize that intrinsic disorder enables Hst5 to maintain functional flexibility, even under physiologically crowded conditions, while local backbone stiffness and electrostatic patterning continue to dictate ensemble properties and functional interactions. Collectively, the interplay among histidine-mediated charge regulation, solvent-mediated interactions, temperature effects, and crowding establishes Hst5 as a tunable system. This environmental sensitivity is central to its ability to interact with membranes, multivalent ions, and solid surfaces, setting the stage for the complex collective phenomena described in subsequent sections.

## Electrostatics, Multivalent Cations, and Dynamic Oligomerization

Beyond the influence of monovalent salts, Hst5 engages in highly specific interactions with multivalent ions, which profoundly modulate both its conformational ensemble and collective behavior. In particular, zinc ions coordinate histidine imidazole groups, thereby promoting reversible, dynamic oligomerization of Hst5 and other histidine-rich IDPs.^21,40,41^ Consistent with its histidine-rich sequence, Hst5 contains several histidine residues, with two specific zinc-binding motifs experimentally identified (Figure 4).^42^ Importantly, interactions between Hst5 and Zn^2+^ are strongly pH dependent. At low pH, both Hst5 and zinc ions carry a high positive charge and interact only weakly (Figure 4A), whereas at near-physiological pH, where the net positive charge of Hst5 is reduced due to partial histidine deprotonation, Zn^2+^ binds specifically to histidine residues. Rather than inducing ordered aggregation, Zn^2+^ binding produces disordered, fluid oligomers in which metal–ligand coordination competes with intrachain electrostatic repulsion and conformational entropy (Figure 4B).^21^ Buffer-independent thermodynamic analyses reveal multiple Zn–Hst5 binding modes and temperature-dependent stoichiometry, consistent with a redistribution of ensemble populations rather than the formation of a distinct aggregated phase. Spectroscopic studies further identify key imidazole donors (e.g., H18/H19) and distinguish Zn^2+^ from Cu^2+^ coordination signatures, while pH-dependent modulation of structure and activity under Zn^2+^/Cu^2+^ reinforces an ensemble-based description of metal-ion regulation.

### Polyvalent Anions, Reentrant Condensation, and Polyelectrolyte Physics

IDPs and disordered regions of globular proteins also play central roles in interactions with, and regulation of, nucleic acids.^44,45^ Their structural flexibility, combined with strong electrostatic attraction to the negatively charged sugar–phosphate backbone of DNA and RNA, enables rapid and adaptive binding.^46^ In the case of Hst5, reentrant condensation has been observed in the presence of the trivalent polyphosphate anion (TPP), closely paralleling classical phenomena from polyelectrolyte physics.^43^ TPP induces a well-defined reentrant condensation window whose onset and dissolution boundaries depend sensitively on the number of arginine residues in the Hst5 sequence (Figure 4C,D). Systematic Arg-to-Lys substitutions demonstrate that these boundaries are governed not only by net charge but also by residue-specific interactions. Combined experimental and coarse-grained simulation studies show that arginine–phosphate interactions, together with charge neutralization and eventual charge inversion at higher anion concentrations, regulate the phase boundaries. These findings underscore the specific role of Arg–phosphate interactions in mediating complexation of IDPs with phosphate-rich biopolymers such as RNA and DNA.^43,47^

### Ion Specificity and Collective Behavior in Disordered Polyelectrolytes

Collectively, these ion-mediated effects place Hst5 near the boundary between dispersed and condensed states, where modest changes in ion valency, concentration, or chemical identity lead to pronounced shifts in ensemble behavior. The balance between intra- and intermolecular interactions, transient oligomer formation, and mesoscale organization is dictated by molecularly resolved ion specificity rather than electrostatics alone. Hst5 therefore exemplifies how an intrinsically disordered polyelectrolyte can generate rich collective behavior, including dynamic oligomerization, reentrant phase transitions, and ion-specific ensemble shifts, without adopting fixed secondary or tertiary structure.

This intrinsic adaptability underpins Hst5′s functional interactions with membranes, solid surfaces, and other biomolecular partners.These behaviors, spanning charge regulation, dynamic oligomerization, and reentrant condensation, are summarized in (Figure 4).

## Interactions at Solid and Membrane Interfaces

In a liquid environment, Hst5 interacts with both solid surfaces and lipid membranes through dynamic, ensemble-based mechanisms governed by intrinsic disorder, electrostatics, and charge regulation by histidine residues.

### Solid Surfaces

On rigid, negatively charged substrates such as silica, Hst5 adsorption is dominated by electrostatic interactions, with both binding strength and surface coverage depending strongly on pH and ionic strength.^4,37^ Monte Carlo simulations and multiscale modeling show that protonation fluctuations of histidine residues enhance surface affinity through charge regulation in response to local electrostatic fields.^4,9,29^ Consistent with these findings, full-length Hst5 exhibits stronger adsorption and higher surface coverage on silica than truncated variants, reflecting cooperative contributions from charged residues distributed along the sequence.^9,37^ Despite strong adsorption, the peptide remains conformationally heterogeneous at the interface and does not adopt a well-defined ordered structure. These molecular mechanisms and their structural consequences at the interface are illustrated in Figure 5, which depicts the context-dependent conformational ensemble of Hst5 upon adsorption to a negatively charged surface.

**Figure 5:**
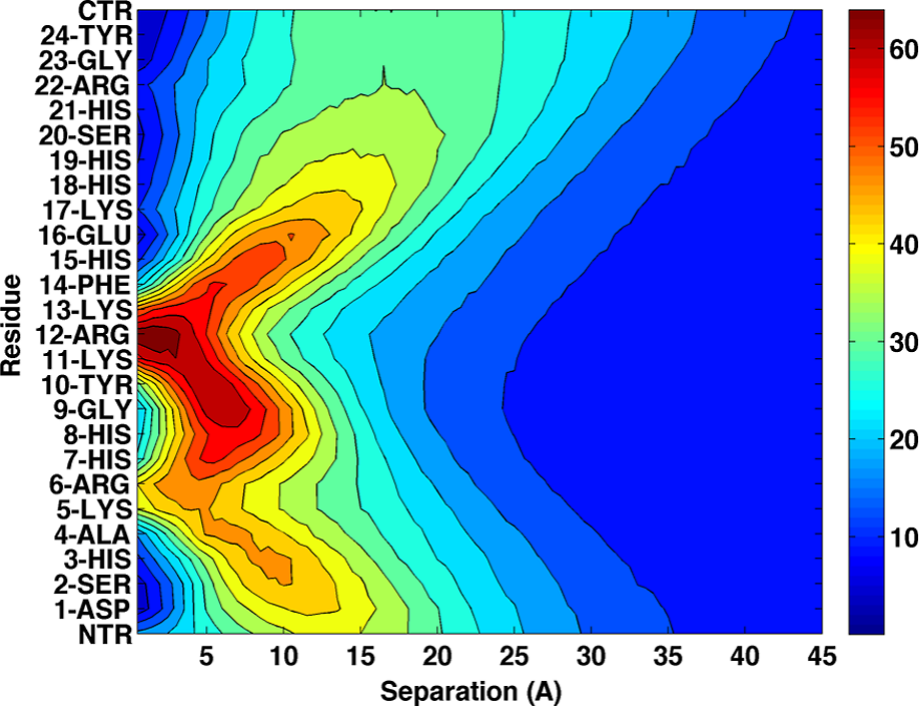
An example of a context-dependent conformational ensemble of Histatin 5 (Hst5) at a charged interface.^4^ Residue-resolved concentration profiles of Hst5 as a function of distance from a negatively charged surface. Positively charged Arg–Lys patches act as dynamic anchoring regions, biasing the ensemble toward surface-bound conformations, while histidine residues contribute short-range stabilization through charge regulation. The data illustrate how adsorption emerges from an adaptive ensemble rather than a single bound structure, underscoring the functional relevance of disorder and charge regulation at biological interfaces. Adapted with permission from ref 4. Copyright 2013 Wiley.

### Lipid Membranes

At fluid lipid bilayers, Hst5 associates primarily through long-range electrostatic interactions and populates a heterogeneous ensemble of surface-bound, partially inserted, and transiently translocated states rather than a single stable membrane-bound conformation.^38^ Neutron reflectometry and QCM-D measurements indicate that histidine content and charge patterning modulate adsorption strength, insertion depth, and membrane perturbation.^36^ Peptide length further influences interaction pathways: at low ionic strength, both truncated variants and full-length Hst5 promote the formation of hydrated, cushioned membrane architectures, whereas deeper insertion and membrane translocation are observed only under conditions that balance electrostatic screening, charge distribution, and chain length, see Figure 6.^20,38^ Membrane crossing most probably, thus proceeds through stochastic sampling of interfacial states rather than via a single well-defined structural intermediate.

**Figure 6:**
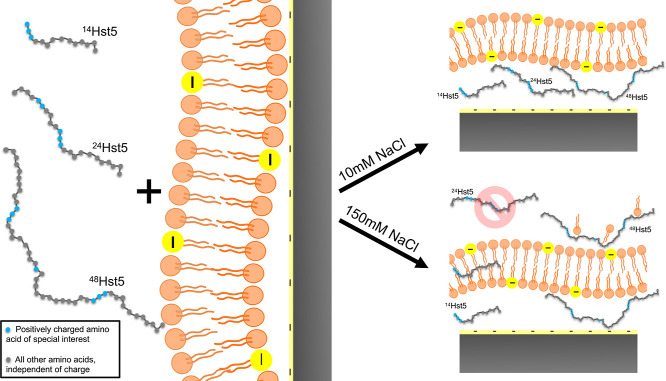
Chain-length–dependent interactions of Histatin 5 (Hst5) variants with supported lipid bilayers. Schematic representation of short (^14^Hst5), medium thus wild-type (^24^Hst5), and long (^48^Hst5) peptides. At low ionic strength (10 mM NaCl), all peptides form a hydrated cushion beneath the bilayer and partially translocate across the membrane. At physiological ionic strength (150 mM NaCl), short peptides (^14^Hst5) maintain membrane insertion and cushion formation, medium peptides (^24^Hst5) show minimal interaction, and long peptides (^48^Hst5) accumulate both below and above the bilayer. Coarse-grained simulations corroborate these experimental trends, highlighting the roles of electrostatic patches and peptide length in mediating adsorption and translocation. Reproduced with permission from ref 20. Copyright 2024 American Chemical Society under license CC-BY 4.0.

## Multiscale Modeling and Methodological Considerations

Accurate modeling of IDPs such as Hst5 has benefited from and contributed to methodological advances in IDP simulation. Conventional force fields often overcompact disordered chains, underestimating R_g_ and ensemble heterogeneity.^24^ ,^31^ ,^48^ ,^49^ In contrast, dispersion-corrected force fields combined with advanced water models such as TIP4P-D reproduce ensemble properties in quantitative agreement with SAXS, DLS, and neutron scattering experiments.^22,23^ These models more faithfully capture global expansion, local backbone stiffness, and PPII content, underscoring the importance of accurately representing protein–solvent interactions.

Coarse-grained modeling and Monte Carlo simulations complement atomistic approaches by enabling exploration of phenomena that remain challenging for fully atomistic models. For Hst5, these methods have elucidated the persistence of extended polyelectrolyte behavior under crowded conditions, reversible zinc-mediated oligomerization, and reentrant condensation in the presence of multivalent ions.^15,21,43^ Monte Carlo simulations combined with ellipsometry have been particularly informative for understanding adsorption dynamics at solid surfaces, revealing how chain length and protonation fluctuations jointly influence surface interactions.^37^

Integrative and machine-learning approaches now enable largely force-field-independent ensemble refinement and sequence-to-ensemble mapping. Multidimensional decomposition of simulation trajectories, combined with experimental restraints from SAXS, neutron scattering, and surface-sensitive techniques, allows subtle sequence-specific effects, such as charge patterning, histidine placement, and chain length, to be quantified and linked to functional behavior.^12,16,20,34,36^ Applied to Hst5 and its histatin siblings, such as Histatin 1 and Histatin 3, these strategies reveal how local stiffness, electrostatics, and multivalent interactions collectively shape ensemble properties and interactions at membranes and surfaces. Complementary atomistic, coarse-grained, and integrative approaches capture the full spectrum of Hst5′s behavior, from local backbone preferences to mesoscale interactions, providing a molecular resolved understanding of IDP function. Hst5 thus serves as a model system for methodological development in IDP simulation,^18,30,50–53^ illustrating how multiscale modeling strategies can converge to predict sequence-encoded ensemble properties and biologically relevant interactions.

## General Physical Principles

Studies of Hst5 illustrate several general principles that characterize IDPs. Functional adaptability arises from its heterogeneous conformational ensemble, enabling the peptide to sample surface-bound, partially inserted, and oligomeric states simultaneously. Charge regulation, driven primarily by histidine protonation, continuously tunes electrostatic interactions with membranes, ions, and solid surfaces, modulating adsorption, translocation, and reversible oligomerization.^20,21,36^

These processes are inherently ensemble-based rather than governed by a single dominant conformation, reflecting the cooperative interplay of chain length, local stiffness, and multivalent interactions. Beyond its biological role as a saliva-derived antimicrobial peptide, its behavior highlights how sequence composition, electrostatics, and solvent interactions collectively determine global expansion, local structure, and collective phenomena. The system exemplifies the power of multiscale modeling and integrative experimental approaches in capturing the rich behavior of disordered proteins, from atomistic flexibility to mesoscale interactions with membranes and surfaces.

## Future Directions and Conclusions

Hst5 continues to serve as a model system for exploring how intrinsic disorder, electrostatics, solvent interactions, and multivalent ions confer functional adaptability in peptides. Future studies will benefit from deeper characterization of specific ion effects, including time-resolved and in situ experiments that track ensemble population shifts under physiologically relevant conditions. Continued refinement of IDP-aware force fields and integrative multiscale modeling approaches will enable predictive mapping from sequence to ensemble behavior, bridging atomistic flexibility with mesoscale interactions at membranes, surfaces, and in crowded environments.

More broadly, Hst5 exemplifies the general physical principles that govern disordered polyelectrolytes: conformational heterogeneity, charge regulation, and ensemble-based binding underpin its versatility. Its study demonstrates how experiments, simulations, and integrative approaches can converge to elucidate complex behaviors in disordered systems, providing both a framework for understanding other IDPs and for advancing theoretical and computational models. As experimental and computational techniques continue to evolve, we foresee thatHst5 will remain of interest to the research community for exploring the interface of biophysics, soft-matter chemistry, and protein design, offering insights relevant to both fundamental science and peptide-based therapeutics.
